# Supplementary material for: Human Peripheral Blood Dendritic Cell and T Cell Activation by Codium fragile Polysaccharide
Source: Mar Drugs. 2020 Oct 27;18(11):535. doi: 10.3390/md18110535 (PMC7692045; doi:10.3390/md18110535)
Supplement: Supplementary file 1 [file marinedrugs-18-00535-s001.pdf]

Supporting Information for

Human peripheral blood dendritic cell and T cell activation by *Codium fragile* polysaccharide

Wei Zhang[1#], Juyoung Hwang[1,2,3#], Hae-Bin Park[1,2,3], Seong-Min Lim[2,3], Seulgi Go[2,3], Jihoe Kim[2,3], Inho Choi[2,3], SangGuan You[4*], and Jun-O Jin[1,2,3*]

[1]Shanghai Public Health Clinical Center, Shanghai Medical College, Fudan University, Shanghai 201508, China
[2]Department of Medical Biotechnology, Yeungnam University, Gyeongsan 38541, South Korea
[3]Research Institute of Cell Culture, Yeungnam University, Gyeongsan 38541, South Korea
[4]Department of Marine Food Science and Technology, Gangneung-Wonju National University, 120 Gangneung Daehangno, Gangneung, Gangwon 210-702, South Korea

* Correspondence: SangGuan You, Department of Marine Food Science and Technology, Gangneung-Wonju National University, 120 Gangneung Daehangno, Gangneung, Gangwon 210-702, South Korea email: umyousg@gwnu.ac.kr and Jun-O Jin, Shanghai Public Health Clinical Center, Shanghai Medical College, Fudan University, Shanghai 201508, China, and Department of Medical Biotechnology, Yeungnam University, Gyeongsan 38541, South Korea, Tel: 82-53-810-3033, E-mail: jinjo@yu.ac.kr

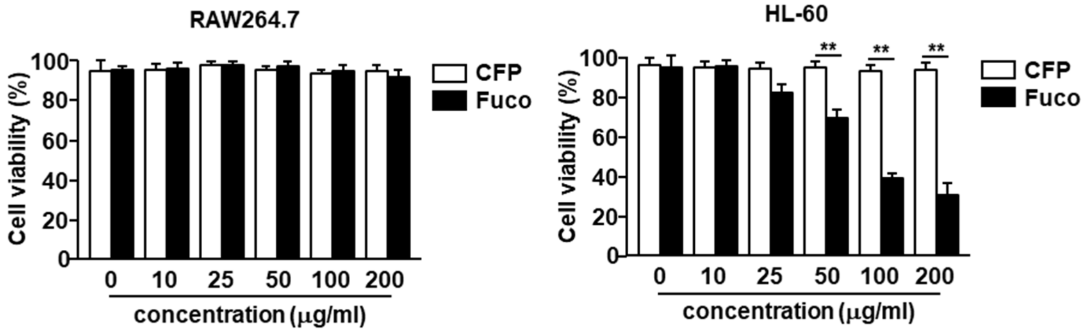

Figure S1. Cytotoxic effect of *Codium fragile* polysaccharide (CFP) and Fucoidan in RAW264.7 and HL-60 cells. RAW264.7 and HL-60 cells were treated with indicated concentration of CFP or fucoidan for 24 hours. The viability of cells was analyzed by MTT assay.

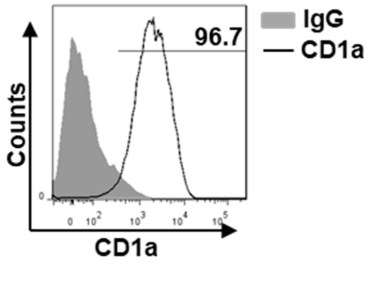

Figure S2. Determination of monocyte-derived dendritic cells (MDDCs) differentiation. Expression of CD1a in the MDDCs was analyzed by flow cytometer.
